# Treatment of infantile fibrosarcoma: A tertiary care center experience

**DOI:** 10.3389/fped.2022.1015185

**Published:** 2022-11-01

**Authors:** Yidi Han, Kai Lian, Dongdong Zhang

**Affiliations:** ^1^ Department of Oncology, Xiangyang No. 1 People's Hospital, Hubei University of Medicine, Xiangyang, China; ^2^ Department of Orthopedics, Xiangyang No. 1 People's Hospital, Hubei University of Medicine, Xiangyang, China; ^3^ Department of Pediatric Hematology/Oncology, Xinhua Hospital Affiliated to Shanghai Jiao Tong University School of Medicine, Shanghai, China

**Keywords:** infantile fibrosarcoma, infant, intergroup rhabdomyosarcoma study group, non-mutilating surgical resection, chemotherapy

## Abstract

**Objective:**

Infantile fibrosarcoma (IFS) is a highly locally aggressive nonrhabdomyosarcomatous soft tissue sarcoma that most commonly occurs in young infants. There exists no standard treatment this lesion due to its rarity. We shared our treatment experience for IFS in this study.

**Methods:**

Patients' record between January 2013 and December 2018 were reviewed and patients with newly diagnosed IFS were included. The clinical characteristics, treatment strategy, treatment-related toxicities and clinical outcome were retrospectively analyzed.

**Results:**

Eleven patients were admitted in our center, including 4 girls and 7 boys, and the median age at diagnosis was 5 months (range 1–23 months). Ten patients achieved complete remission (CR) after the completion of initial treatment. The main short-term adverse effects was myelosuppression. Three patients experienced relapse, including two patients with local progression and one patient with distant metastasis. After a median follow-up of 3.5 years (range 1.5–7 years), 9 patients were alive and 2 patients died. The 3-year overall survival (OS) rate was 93.5% (95% CI 83.7–98.2).

**Conclusion:**

We formulated the treatment strategy according to group grade and the experience from previous studies, which may be effective and feasible for the treatment of IFS.

## Introduction

Infantile fibrosarcoma (IFS) is a rare type of non-rhabdomyosarcoma soft tissue tumor that is currently classified as “intermediate malignancy”. IFS is the most common soft tissue tumor in children less than 2 years of age (1). It can be seen at birth or during early childhood, in some cases, it can also be diagnosed in children up to 4 years of age (2). IFS is usually presents as a rapidly growing, non-tender, poorly circumscribed mass with low incidence of metastases (3). Previously, scientists have suggested that IFS is linked with cytogenetic translocation t (12; 15) (p13; q25), resulting in *ETV6–NTRK3* gene fusion. Besides this, some other translocations, such as *EML4-NTRK3*, *TPM3-NTRK1*, *LMNA-NTRK1* and *BRAF* intragenic deletions, have also been observed in IFS (4). IFS has a distinct pathology, cytogenetic profile, and clinical outcome compared with adult fibrosarcoma although they shared similar histology (5). Overall, IFS has a satisfactory prognosis, more that 80% of patients are potentially cured and the reported 10-year survival rate is 90% (3, 6).

Only a limited number of IFS can regress spontaneously without treatment (7). To date, surgical extirpation is considered a curable treatment approach for IFS. However, IFS commonly has a large tumor size at the time of presentation, which makes complete resection impossible (8). Therefore, conservative surgery so as to avoid functional damage remains the mainstay treatment for IFS (9). Of special note, about 48%–62% of primary tumors are unresectable and require a multidisciplinary strategy, including preoperative cytoreductive treatment and local radiotherapy in a particular situation (3, 10, 11). Radiotherapy application is limited because of its long-term complications and sequelae. Considering the chemosensitivity of IFS, preoperative chemotherapy can be used in inoperable patients, and delayed conservative surgery or complete resection may be performed when tumor shrinkage is achieved; postoperative chemotherapy has been recommended as the first-line treatment for patients with macroscopic residual disease to decrease the local recurrence (10). Although various studies have reported efficacy of many combinations, the standardized chemotherapy guidelines have not yet been well defined. In consideration of the very young age of patients, the optimal choice should be made after weighing between the short and long-term toxicities and the need to achieve effectiveness. We report our experiences in the clinical management of IFS in this retrospective study.

## Patients and methods

### Patients

Patients aged from birth to 2 years, with newly diagnosed IFS, and previously untreated, were included between January 2013 and December 2018. The medical records were retrospectively reviewed and analyzed for demographic details and clinical outcomes. The *ETV6-NTRK* status was detected by fluorescence immunofluorescence *in situ* hybridization. The diagnosis of IFS was based on age at diagnosis, radiology, and molecular pathology. This study was authorized by Hubei University of Medicine with approval number XH2021006.

### Treatment, toxicities and response

Patients were classified into four groups according to the Intergroup Rhabdomyosarcoma Study Group (IRSG) system (Table 1).

**Table 1:** Clinical group and treatment strategy for IFS in this study.

Group	Definition
I	Localized lesions with microscopically complete resection without regional lymph node metastasis
II	Localized lesions with microscopically incomplete resection or regional lymph nodal spread
III	Localized lesions with macroscopic residual disease
IV	Distant metastases

Briefly, the National Cancer Institute Common Terminology Criteria for Adverse Events version 4.0 was used for the grade of adverse events (12). Treatment response was assessed by the Modified Response Evaluation Criteria in Solid Tumors (RECIST) (13). Overall survival (OS) and event-free survival (EFS) were estimated by the Kaplan-Meier curve. OS was calculated from the date of initial diagnosis to the date of last follow-up (including death), EFS was calculated from the date of diagnosis to the date of first event.

## Results

### Patients

The detailed clinical characteristics, treatment response and clinical outcomes of patients with IFS are listed in Table 2. A total of 11 patients were enrolled, including 4 girls and 7 boys. The clinical diagnosis was confirmed by pathologists, and all the patients had positive *ETV6–NTRK3* translocation*.* The median age at diagnosis was 5 months (range 1–23 months). The most common primary site was limbs (54%), and the second most common site was the trunk. The initial tumor size was large in infants, ranging from 2.5 cm to 16 cm.

**Table 2:** Clinical characteristics, treatment-relate toxicities and clinical outcome of patients with IFS.

Patient	Age (Month)	Sex	Primary site	Tumor size	Histopathology (*ETV6-NTRK*)	Group	Treatment	Response	Toxicities	Outcome
1	5	Female	Abdomen	10.0* 8.0* 8.0 cm	Positive	III	Surgery plus 13 cycles of VAC regimen	CR	Grade IV myelosuppression, Grade II vomiting	Alive (7 years)
2	23	Male	Forearm	5.0*3.0*2.6 cm	Positive	III	Initial treatment: Surgery plus 11 cycles of VAC regimen	Lung metastasis after 1 years of CR	Grade IV myelosuppression	Dead (3 years later)
						IV	Salvage therapy: 6 cycles of VAID and ICE regimen and Radiotherapy (pulmonary radiation, 12 Gy)	Lung metastasis achieved PD after 6 months		
3	4	Male	Abdomen	10.0*8.0*6.5 cm	Positive	III	Surgery plus 11 cycles of VAC regimen	CR	Grade IV myelosuppression	Alive (6 years)
4	3	Female	Sacrococcygeal region	4.0*4.0*5.0 cm	Positive	III	Surgery plus 13 cycles of VAC regimen	CR	Grade IV myelosuppression	Alive (4 years)
5	2	Male	Arm	6.0* 2.0*2.0 cm	Positive	I	4 cycles of preoperative VAC regimen followed surgery (R0 resection)	CR	Grade III myelosuppression, elevated liver enzymes	Alive (5 years)
6	5	Male	Pelvic	9.0*14.0 *16.0 cm	Positive	III	Initial treatment: 8 cycles of VAC regimen	Local progression and lung metastasis after 6 months	Grade IV myelosuppression, febrile neutropenia, hydronephrosis, pulmonary infection,	Dead (1.5 years later)
						IV	Salvage therapy: resection of lung metastasis, 3 cycles of VAID regimen and radiotherapy (abdominal radiation, 45 Gy)	Local progression (hydronephrosis) after 2 months		
7	2	Male	Preauricular region	2.5*2.0*2.0 cm	Positive	II	Surgery followed observation	Local relapse after 9 months	Grade IV myelosuppression, Grade II vomiting	Alive (3.5 years)
						II	Reoperation plus 13 cycles of VAC regimen	CR		
8	1	Female	Arm	4.3*2.0 *1.4 cm	Positive	III	Surgery plus 11 cycles of VAC regimen	CR	Grade IV myelosuppression	Alive (3.5 years)
9	1	Male	Foot	1.4*0.6 *1.0 cm	Positive	II	Surgery plus 4 cycles of VAC regimen	CR	Grade II myelosuppression	Alive (3.5 years)
10	2	Male	Forearm	7.0*3.5*2.0 cm	Positive	III	Surgery plus 13 cycles of VAC regimen	CR	Grade IV myelosuppression	Alive (3.5 years)
11	7	Female	Leg	3.7*2.3*4.7 cm	Positive	III	Surgery plus 13 cycles of VAC regimen	CR	Grade IV myelosuppression	Alive (5 years)

Note: All the regimens were administrated at an interval of 21 days.

VAC regimen: vincristine, 0.05 mg/kg, d1; actinomycin-D: 0.045 mg/kg, d1; cyclophosphamide: 40 mg/kg, d1.

VAID regimen: vincristine:0.05 mg/kg, d1; doxorubicin 1.0 mg/kg,d2; cyclophosphamide: 10 mg/kg, d2–4, cisplatin: 3.0 mg/kg, d1.

VIE regimen: vincristine:0.05 mg/kg d1, ifosfamide: 50 mg/kg, d2–6; etoposide: 3.33 mg/kg, d2–6.

### Treatment and related toxicities

Only one patient received neoadjuvant chemotherapy followed by complete resection. Nine patients underwent surgery followed by adjuvant chemotherapy. One patient (patient 6#) with an inoperable tumor was progressed during the chemotherapy and lost the chance to undergo an operation. One patient (patient 7#) in Group II treated with surgery without adjuvant chemotherapy experienced local recurrence after 9 months, and complete remission was achieved after reoperation and adjuvant chemotherapy. Two patients received local radiotherapy after the recurrence. Patient 2 received pulmonary radiation (12 Gy, 8 fractions) and patient 6 received abdominal radiation (45 Gy, 25 fractions). Two patients died due to disease progression. One patient (patient 2#) died of severe pulmonary infection and respiratory failure caused by lung metastasis three years later. One patient (patient 6#) died from hydronephrosis, pulmonary infection, multi-organ dysfunction caused by giant tumor pressure, and lung metastasis. The common side effects were myelosuppression and gastrointestinal reaction.

### Treatment response

**Figure 1:**
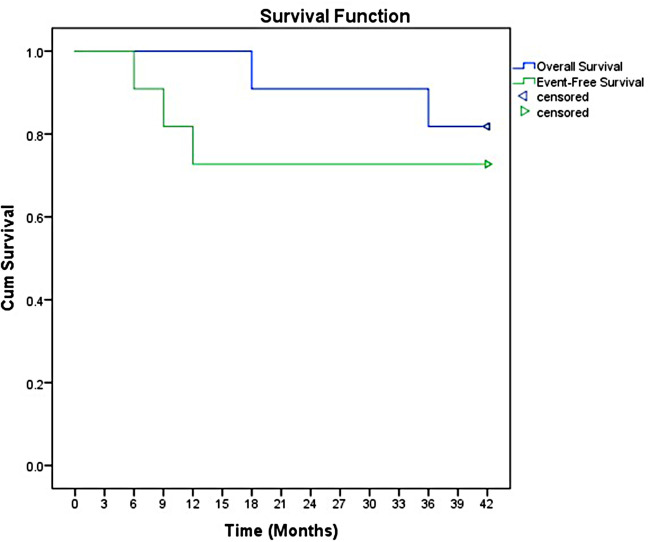
The event-free survival and overall survival of IFS.

The overall response rate was 90.9%, including ten patients achieved complete remission (CR). Only two patients received salvage regimen. However, the salvage regimens seemed to have no effect in delaying disease progression. The overall objective remission rate (ORR) was 81.8% (9/11 cases). After a median follow-up of 3.5 years (range 1.5 to 7 years), the 3-year event-free survival (EFS) and OS were 78.5% and (95% CI 57.8–88.7) and 93.5% (95% CI 83.7–98.2), respectively (Figure 1).

## Discussion

To date, the precise definition of IFS remains debatable. Since many rapidly progressive infantile soft tissue tumors, such as hemangiopericytomas and primary myxoid mesenchymal tumor of infancy, shared a similar histologic feature with IFS (14), the special *ETV6–NTRK3* translocation could be used for the diagnosis of IFS. Notably, some IFSs have other translocation as previously described. Moreover, the *ETV6–NTRK3* translocation could also be detected in some other pediatric tumors, such as mesoblastic nephroma and high-grade glioma (15). Therefore, diagnosis should be based on the age of onset, clinical presentation, histologic features, and molecular characteristics.

A few case reports have shown that IFS had the possibility of spontaneous regression and recommended that clinicians should weigh the risk between treatment and watchful waiting (7). In our opinion, the “wait and watch” strategy might be considered when patients are <6 months of age, parents refuse surgery, primary tumor size is <5 cm and is not a life-threatening site.

Although neoadjuvant chemotherapy has played a key role in controlling the tumor size in inoperable patients, surgery remains the cornerstone of treatment for IFS. Adjuvant chemotherapy has been recommended for patients in Group III to reduce the local recurrence (3, 10). However, whether postoperative adjuvant chemotherapy should be given to Group II patients remains controversial. The European Pediatric Soft Tissue Sarcoma Study Group (EpSSG) demonstrated that adjuvant chemotherapy was not necessary in patients with a microscopically incomplete resection as the local recurrence rate was 12.5% (1/8 cases) (10). A retrospective case review indicated that patients in Group II exclusive chemotherapy had a 16.6% (2/12 cases) recurrence rate (16). All these results indicated patients in Group II had a relatively low recurrence rate after surgery without adjuvant chemotherapy; however, this conclusion was drawn from a small sample. Moreover, whether adjuvant chemotherapy after surgery could be beneficial to patients in Group II still needs to be studied further.

To reduce the gonadal and mutagenic toxicity of an alkylating agent and the cardiac toxicity of anthracycline, an alkylating agent–free and anthracycline-free regimen (vincristine plus dactinomycin, VA) was recommended as the first line treatment for an inoperable tumor (3). In our study, patients in Group III received at least 8 cycles of the VAC regimen; however, patients in the European study received only 6 cycles of the VA regimen or 4 cycles of the VAC regimen. Although the ORR in our study was similar to that in the EpSSG (81.7% vs. 71%), it seemed that some patients in our study might have been overtreated. Furthermore, considering the long-term toxicities of cyclophosphamide, we believed that the VA regimen was more suitable for children with IFS.

Radiotherapy and mutilating surgery might be considered after the failure of salvage therapies (10). Radiotherapy was previously administered at inoperable axial primary sites (16). In our study, radiotherapy after the failure of salvage therapies had no impact on controlling the progression. In view of the side effects of radiotherapy on children's growth and development, it should be recommended with caution on an individual basis.

NTRK inhibitor (TRKi) showed a rapid, complete, and sustained response in patients with IFS who were ETV6-NTRK positive after the resistance to chemotherapy (17). Some clinical trials indicated that a NTRK inhibitor exhibited a good response with limited toxicities and could be used as neoadjuvant treatment for inoperable tumors (18, 19); it was also recommended as complementary therapy after the failure of salvage therapies before mutilating surgery for advanced IFS (20), but an international consensus was not reached. Currently, TRKi is recommended for patients with an unresectable tumor and conventional chemotherapy failure, or patients with metastatic disease (20). The NTRK protein played an important role in the early development of brain; considering the fact that the long-term neurodevelopmental toxicity in very young children was still unclear, TRKi is not chosen as the upfront treatment for IFS. No patients have been treated with an TRKi in this study because the TRKi were not approved in China.

In conclusion, non-mutilating surgery was the mainstay treatment for IFS. A “wait and see” strategy was applied in patient in Group I after surgery. In patients with a positive surgical margin, postoperative chemotherapy was beneficial for decreasing the local recurrence rate, even in patients with a microscopically incomplete resection. The VA regimen could be recommended as an upfront treatment for IFS patients after surgery in Group II-III. For patient in Group IV, systemic chemotherapy was conventionally given after local resection or biopsy alone. TRKi may be recommended for the treatment of patients with an unresectable tumor or metastatic disease, but further studies are needed to evaluate the efficacies and long-term toxicities.

## Data Availability

The original contributions presented in the study are included in the article/Supplementary Material, further inquiries can be directed to the corresponding author/s.
